# Inferring steady state single-cell gene expression distributions from analysis of mesoscopic samples

**DOI:** 10.1186/gb-2006-7-12-r119

**Published:** 2006-12-14

**Authors:** Jessica C Mar, Renee Rubio, John Quackenbush

**Affiliations:** 1 Department of Biostatistics, Harvard School of Public Health, Huntington Avenue, Boston, Massachusetts 02115, USA; 2 Department of Biostatistics and Computational Biology, Dana-Farber Cancer Institute, Binney St, Boston, Massachusetts 02115, USA; 3 Department of Cancer Biology, Dana-Farber Cancer Institute, Binney St, Boston, Massachusetts 02115, USA

## Abstract

**Background:**

A great deal of interest has been generated by systems biology approaches that attempt to develop quantitative, predictive models of cellular processes. However, the starting point for all cellular gene expression, the transcription of RNA, has not been described and measured in a population of living cells.

**Results:**

Here we present a simple model for transcript levels based on Poisson statistics and provide supporting experimental evidence for genes known to be expressed at high, moderate, and low levels.

**Conclusion:**

Although the model describes a microscopic process occurring at the level of an individual cell, the supporting data we provide uses a small number of cells where the echoes of the underlying stochastic processes can be seen. Not only do these data confirm our model, but this general strategy opens up a potential new approach, Mesoscopic Biology, that can be used to assess the natural variability of processes occurring at the cellular level in biological systems.

## Abstract 2

A simple model for assessing transcript levels based on Poisson statistics is proposed and validated by estimating the variance on gene expression levels as a function of the number of cells surveyed.

## Background

In the study of biological processes, most of our observations are based on measurements made on a macroscopic scale, such as a piece of tissue or the collection of cells in a tissue culture dish, while the processes themselves are driven by events that occur at a microscopic scale representing events within each individual cell. The paradox here is that, macroscopically, biological processes often seem deterministic and are driven by what we observe as the average behaviour of millions of cells, but microscopically we expect the biology, driven by molecules that have to come together and interact in a complex environment, to have a stochastic component. Indeed, studies of transcriptional regulation at the single cell level have uncovered examples of non-uniform behaviour of gene expression in genetically identical cells. Levsky *et al*. [1] were among the first to profile gene expression levels in single cells and their results provided direct evidence of variable expression patterns in otherwise identical cells. Ozbudak *et al*. [2] quantified the direct effect that fluctuations in molecular species had on the variation of gene expression levels in isogenic cells. By independently modifying transcription and translation rates of a single fluorescent reporter protein, they were able to observe the downstream effects this had on protein expression. From these experiments, the authors were able to conclude that protein production occurs in sharp, random bursts. This was further explored by Cai *et al*. [3], who developed a microfluidic-based assay to observe proteins being produced in real-time inside a living cell. They provide experimental proof that proteins are expressed in bursts and demonstrate that the number of molecules per burst follows an exponential distribution. While this represents an important advance, the mechanisms governing this behaviour are not yet fully known and building relevant models requires some knowledge of each of the basic processes involved in the pathway from DNA to RNA to protein.

Over the past 30 years, numerous mathematical models of stochastic gene expression have been proposed [4,5]. Rao *et al*. [6] outline some of the most general of these approaches and show how they have been improved into more sophisticated models by various researchers. One of the most basic models is a stochastic differential equation that monitors the production rate of a molecular species (DNA, RNA or protein). This is simply a differential equation with a random noise term and a stochastic process or random variable that accounts for the amount of molecule available at a given time. Such models representing components of a particular system are then mathematically coupled to predict the output levels of genes, mRNAs, and proteins produced inside a single cell. A basic question that remains to be fully explored, however, is whether evidence of these stochastic elements exists and if gene expression is truly a stochastic process? With respect to RNA, the answers to these questions have, thus far, been elusive. The problem is that nearly the entirety of RNA expression data come from large samples where the observed gene expression levels are an ensemble average over millions of cells. However, what we ultimately want to understand is the distribution of RNA levels in individual cells, something that has been difficult to measure. Here we propose a simple but elegant solution to this problem, which we refer to as 'Mesoscopic Biology'. In this approach, we conduct experiments between the microscopic and macroscopic levels, working with a small but finite number of cells where measurements can be easily made but where evidence of stochastic processes operating at a cellular level are not lost through the biological averaging that occurs when in large samples.

As a demonstration of the power of the mesoscopic approach, we demonstrate for the first time that RNA transcript levels obey Poisson statistics for genes expressed at various levels within the cell. We begin by modelling mRNA copy number within a cell as a Poisson random variable and derive an analytical solution that captures the randomness in gene expression, manifested as an increase in measured biological variability as we decrease the number of cells assayed in a particular experiment. Using a dilution series experiment and measuring the expression of nine genes using quantitative real-time RT-PCR (qRT-PCR), we validate the model and provide estimates of the average expression level for each.

## Results and discussion

### Theoretical model

The Poisson distribution is a mathematical function that assigns a probability to measuring a certain number of events within a defined time frame. The Poisson distribution is similar to the Normal or Gaussian distribution - the familiar 'bell curve' - except that, while the latter is centered symmetrically about its mean, the Poisson distribution is skewed to the right, and its 'mass' is concentrated somewhere on a scale between zero and infinity.

Poisson statistics have a long history of being used to model count data and counting processes [7] where there is a fixed lower limit in the count (zero). Consequently, a natural assumption is that the number of mRNA copies inside a single cell follows a Poisson distribution. If we view a whole tissue as being made up of *N *cells of the same type, then the corresponding expression levels for each gene, represented as the number of mRNA copy numbers in each cell, can be cast as a sample of *N *independent, identically distributed Poisson random variables; note this is a simplifying assumption that we have made for the purposes of modelling mRNA counts. Assigning a probability distribution function to mRNA copy numbers allows us to capture the stochastic nature of the underlying transcriptional process while providing a means to estimate overall properties and to make inferential statements about how these properties behave as we change the number of cells under analysis. In particular, such a statistical model allows us to estimate parameters, such as the average copy number per cell for each gene-specific transcript. Specifically, we expect the average gene expression to behave like a Normal random variable as the size of the biological sample (that is, the number of cells, *N*) grows. This result follows from the Central Limit theorem and gives us a way to derive analytical statements about how the variability in gene expression will change with sample size.

Specifically, suppose that each cell makes, on average, a certain number of copies (say λ) of a particular gene. In this case, the probability that a cell produces exactly *x *copies of a gene is given by the standard form of the Poisson probability distribution:

P(X=x)=λxe−λx!
 MathType@MTEF@5@5@+=feaafiart1ev1aaatCvAUfeBSjuyZL2yd9gzLbvyNv2Caerbhv2BYDwAHbqedmvETj2BSbqee0evGueE0jxyaibaiKI8=vI8tuQ8FMI8Gi=hEeeu0xXdbba9frFj0=OqFfea0dXdd9vqai=hGuQ8kuc9pgc9s8qqaq=dirpe0xb9q8qiLsFr0=vr0=vr0dc8meaabaqaciGacaGaaeqabaqadeqadaaakeaacaWGqbGaaiikaiaadIfacqGH9aqpcaWG4bGaaiykaiabg2da9maalaaabaacciGae83UdW2aaWbaaSqabeaacaWG4baaaOGaamyzamaaCaaaleqabaGaeyOeI0Iaeq4UdWgaaaGcbaGaamiEaiaacgcaaaaaaa@41A4@

If we let X¯
 MathType@MTEF@5@5@+=feaafiart1ev1aaatCvAUfeBSjuyZL2yd9gzLbvyNv2Caerbhv2BYDwAHbqedmvETj2BSbqee0evGueE0jxyaibaiKI8=vI8tuQ8FMI8Gi=hEeeu0xXdbba9frFj0=OqFfea0dXdd9vqai=hGuQ8kuc9pgc9s8qqaq=dirpe0xb9q8qiLsFr0=vr0=vr0dc8meaabaqaciGacaGaaeqabaqadeqadaaakeaaceWGybGbaebaaaa@3422@ denote the average gene expression across the total cell population, then for a large number of cells *N*, the average gene expression X¯
 MathType@MTEF@5@5@+=feaafiart1ev1aaatCvAUfeBSjuyZL2yd9gzLbvyNv2Caerbhv2BYDwAHbqedmvETj2BSbqee0evGueE0jxyaibaiKI8=vI8tuQ8FMI8Gi=hEeeu0xXdbba9frFj0=OqFfea0dXdd9vqai=hGuQ8kuc9pgc9s8qqaq=dirpe0xb9q8qiLsFr0=vr0=vr0dc8meaabaqaciGacaGaaeqabaqadeqadaaakeaaceWGybGbaebaaaa@3422@ follows a Normal distribution with mean λ and variance λN
 MathType@MTEF@5@5@+=feaafiart1ev1aaatCvAUfeBSjuyZL2yd9gzLbvyNv2Caerbhv2BYDwAHbqedmvETj2BSbqee0evGueE0jxyaibaiKI8=vI8tuQ8FMI8Gi=hEeeu0xXdbba9frFj0=OqFfea0dXdd9vqai=hGuQ8kuc9pgc9s8qqaq=dirpe0xb9q8qiLsFr0=vr0=vr0dc8meaabaqaciGacaGaaeqabaqadeqadaaakeaadaWcaaqaaiabeU7aSbqaaiaad6eaaaaaaa@35C4@. This simple model lets us analytically infer how biological variability will behave within a population of *N *'identical' cells and make predictions that can be experimentally verified. Note that in any measurement, there are systematic sources of error (or variability) and those that represent the true distribution of the quantity we measure within the population. Biological variability refers to the 'noise' or variability specific to the biological system under study. Imagine that we were somehow able to control for all types of experimental and technical noise in our measurements, then the remaining variation would be a result of naturally occurring biological variability. The standard deviation of blood pressure measurements is an example of biological variability in a population of individuals. The variation in the number of transcripts in each cell is the biological variation we are trying to model.

### Simulations: visualizing the model

**Figure 1:**
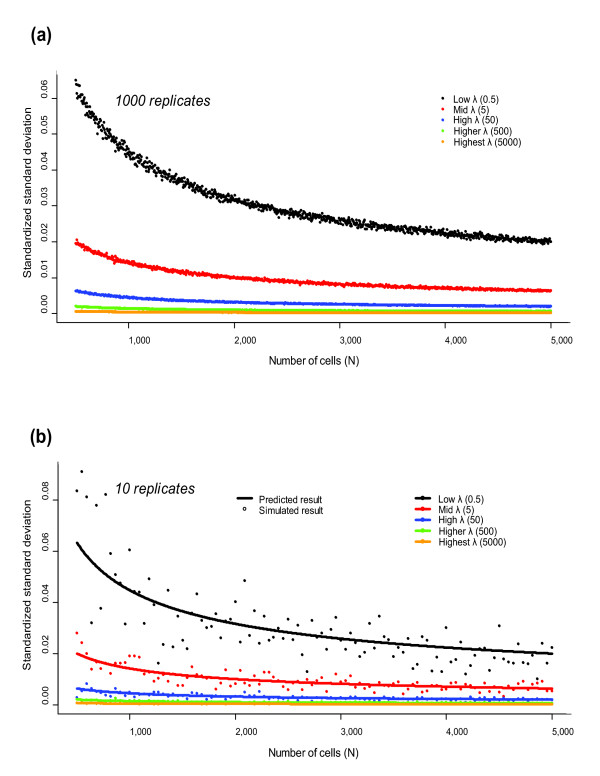
**(a) **Trends in variability as the size of the cell population increases are shown for five different levels of λ, representing 'high', 'medium' and 'low' levels of gene expression. Variability is shown by the standardized standard deviation (a measure of variance) of simulated gene expression values calculated across 1,000-fold replicated populations of cells, and has been standardized by average gene expression. The standardized variance is another way of showing how the variance changes with respect to the number of cells in our virtual population. Higher values will always be associated with higher variance so we standardized by the mean value to see the true behavior of the system. As we expect the variance to follow the analytic solution λN
 MathType@MTEF@5@5@+=feaafiart1ev1aaatCvAUfeBSjuyZL2yd9gzLbvyNv2Caerbhv2BYDwAHbqedmvETj2BSbqee0evGueE0jxyaibaiKI8=vI8tuQ8FMI8Gi=hEeeu0xXdbba9frFj0=OqFfea0dXdd9vqai=hGuQ8kuc9pgc9s8qqaq=dirpe0xb9q8qiLsFr0=vr0=vr0dc8meaabaqaciGacaGaaeqabaqadeqadaaakeaadaWcaaqaaiabeU7aSbqaaiaad6eaaaaaaa@35C4@, standardizing the variance by the mean (for a Poisson random variable, the mean is also λ) will give overall data that decays according to 1N
 MathType@MTEF@5@5@+=feaafiart1ev1aaatCvAUfeBSjuyZL2yd9gzLbvyNv2Caerbhv2BYDwAHbqedmvETj2BSbqee0evGueE0jxyaibaiKI8=vI8tuQ8FMI8Gi=hEeeu0xXdbba9frFj0=OqFfea0dXdd9vqai=hGuQ8kuc9pgc9s8qqaq=dirpe0xb9q8qiLsFr0=vr0=vr0dc8meaabaqaciGacaGaaeqabaqadeqadaaakeaadaWcaaqaaiaaigdaaeaacaWGobaaaaaa@34CB@. We chose to represent the standardized standard deviation (the square root transformation of the variance) because this quantity will follow the analytic solution λN/λ=1Nλ
 MathType@MTEF@5@5@+=feaafiart1ev1aaatCvAUfeBSjuyZL2yd9gzLbvyNv2Caerbhv2BYDwAHbqedmvETj2BSbqee0evGueE0jxyaibaiKI8=vI8tuQ8FMI8Gi=hEeeu0xXdbba9frFj0=OqFfea0dXdd9vqai=hGuQ8kuc9pgc9s8qqaq=dirpe0xb9q8qiLsFr0=vr0=vr0dc8meaabaqaciGacaGaaeqabaqadeqadaaakeaadaWcgaqaamaakaaabaWaaSaaaeaacqaH7oaBaeaacaWGobaaaaWcbeaaaOqaaiabeU7aSjabg2da9maalaaabaGaaGymaaqaamaakaaabaGaamOtaiabeU7aSbWcbeaaaaaaaaaa@3C26@ and, therefore, we can represent different curves for different values of λ. **(b) **Trends in variability as the cell population size changes are highlighted for a simulated example with a lower (ten-fold) degree of replication. The standardized variance of simulated gene expression values is shown by dots, and the standardized variance given by our analytical model is shown by the bold line. This suggests that, even with a moderate number of replicates, we should be able to observe a distinct effect dependent on the gene expression level.

To illustrate the expected behaviour of such a model, we performed simulations of different total cell populations (a range of *N *= 500 to *N *= 5,000 in increments of 5) and assumed representative genes with low, medium, and high levels of expression (λ = 0.5, 5, 50, 500, 5,000). For each value of λ, we generated 1,000 repeated simulations, and for each *N*, we calculated both the average expression and its variance and plotted those as a function of the number of cells (Figure 1a); similar results were also derived for a more realistic situation involving 10 repeated measures (Figure 1b). As one would expect from the Central Limit theorem, the variability grows as the number of cells sampled decreases. The reason for this is simple: for small numbers of cells, we face the possibility of occasionally choosing a set that expresses a particular gene at unusually high or low levels simply due to sampling, while for large numbers of cells such variations 'average out' and hide any anomalous behaviour. The analytic solution, λN
 MathType@MTEF@5@5@+=feaafiart1ev1aaatCvAUfeBSjuyZL2yd9gzLbvyNv2Caerbhv2BYDwAHbqedmvETj2BSbqee0evGueE0jxyaibaiKI8=vI8tuQ8FMI8Gi=hEeeu0xXdbba9frFj0=OqFfea0dXdd9vqai=hGuQ8kuc9pgc9s8qqaq=dirpe0xb9q8qiLsFr0=vr0=vr0dc8meaabaqaciGacaGaaeqabaqadeqadaaakeaadaWcaaqaaiabeU7aSbqaaiaad6eaaaaaaa@35C4@, was superimposed on the simulated data in Figure 1 to demonstrate how it captures this variability. Because the validity of this analytical solution is based on asymptotic assumptions, the fit improves as the number of replicates increases. Nevertheless, even with ten replicates, we see that the analytical solution does an adequate job of explaining the overall trend of biological variability as a function of the number of cells in the sample.

### Experimental validation

**Figure 2:**
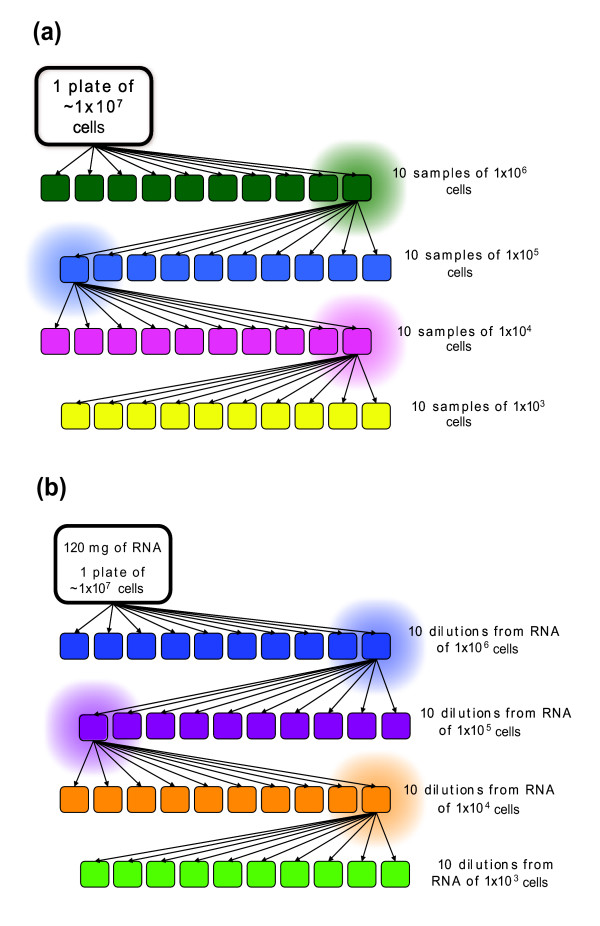
**(a) **Schematic outline of the cell culture serial dilution performed to validate our analytical model. A plate of SW620 cell culture was divided into 10 samples, each containing approximately 1 × 10^6 ^cells. One of these samples was selected at random and divided into a further 10 samples. The cell culture dilution scheme continues until 10 samples of 1 × 10^3 ^cells are achieved; there were a total number of 37 cell culture samples in our experiment. **(b) **Schematic outline of the RNA serial dilution that was used to control and estimate the error in our experimental data. RNA was first extracted from a plate of SW620 cell culture, then divided into 10 identical samples. One of these samples was selected at random to be further divided into 10 samples. A set of 37 controls corresponding to the cellular dilutions was obtained and used to estimate systematic variation in this analysis.

A model without validation is of little use. Consequently, we conducted a series of qRT-PCR experiments to measure the expression of nine genes in epithelial cells derived from the human SW620 colon cancer cell line. Cells were harvested from two plates of cell culture that each contained approximately 1 × 10^7 ^cells. For the first plate, we performed a serial dilution as shown in Figure 2a. The initial culture was diluted into 10 samples, each containing approximately 1 × 10^6 ^cells; one of these was selected at random and diluted into a second set of 10 samples (10 replicates of approximately 1 × 10^5 ^cells). This process was repeated twice more to produce sets of samples containing approximately 1 × 10^4 ^and 1 × 10^3 ^cells. From each of the 37 dilution samples, RNA was extracted as described in the methods. As a means of estimating and controlling for experimental error due to working with small RNA concentrations and its effect on qRT-PCR detection, we first extracted RNA from the second plate and performed identical serial dilutions on the RNA (Figure 2b).

We targeted nine genes for qRT-PCR validation representing 'high,' 'medium,' and 'low' expression levels (Table 1), those encoding: β-actin (*ACTB*), glyceraldehyde-3-phosphate dehydrogenase (*GAPDH*); discoidin domain receptor family, member 1 (*DDR1*); GNAS complex locus (*GNAS*); pinin, desmosome associated protein (*PNN*); phosphoinositide-3-kinase (*PIK3*); ATP synthase, H+ transporting, mitochondrial F0 complex, subunit G (*ATP5L*); polymerase (DNA directed), eta (*POLH*); zinc finger, CCHC domain containing 7 (*ZCCHC7*). We based our gene selection based on 'known' levels of expression (*ACTB *and *GAPDH *are oft-cited examples of highly expressed genes and *PIK3 *is known to be expressed at low levels) as well as expression levels measured from a third, independent cell culture sample using the Affymetrix Human Genome U133 Plus 2.0 GeneChip™. qRT-PCR primers were designed from exonic sequence using Primer3 from the Whitehead Institute [8] and relative expression levels were then verified for these 9 genes in each of the 37 cell dilutions and 37 control RNA dilutions.

Any measured value ultimately represents a convolution of the true signal and an error associated with the measuring process. For macroscopic samples, separating out these two sources is typically straightforward, especially in the presence of a strong and genuine signal and low relative levels of background noise. When working with small samples, however, these two sources are more tightly entwined and the de-convolution process is a more challenging exercise. In assessing gene expression measurements obtained using qRT-PCR, the most significant source of error is the Monte Carlo effect [9], which can produce anomalies observed due to differences in amplification efficiencies between individual RNA species, particularly when a complex RNA sample is being used. In our analysis, the RNA dilution series was designed to allow us to estimate this effect as each pool at a particular dilution level should have the same approximate transcript density as samples in the experimental tissue culture dilution series. When considering biological and experimental sources of variability, it is reasonable to assume that these sources are both independent and, therefore, additive. Hence we can estimate the gene expression levels in our culture dilution by estimating the experimental variability from the RNA dilution series data and subtracting it from the culture dilution series data.

The raw qRT-PCR data were quantified using ABI Prism 7900HT SDS software (version 2.2.2, Applied Biosystems, Foster City, CA, USA). Estimates of experimental error at each dilution series step came from the within-sample variance of the gene expression measures (qRT-PCR quantification values) from the RNA dilution (σEXP2
 MathType@MTEF@5@5@+=feaafiart1ev1aaatCvAUfeBSjuyZL2yd9gzLbvyNv2Caerbhv2BYDwAHbqedmvETj2BSbqee0evGueE0jxyaibaiKI8=vI8tuQ8FMI8Gi=hEeeu0xXdbba9frFj0=OqFfea0dXdd9vqai=hGuQ8kuc9pgc9s8qqaq=dirpe0xb9q8qiLsFr0=vr0=vr0dc8meaabaqaciGacaGaaeqabaqadeqadaaakeaacqaHdpWCdaqhaaWcbaGaamyraiaadIfacaWGqbaabaGaaGOmaaaaaaa@3855@). An estimate of the true biological variability σBIO2
 MathType@MTEF@5@5@+=feaafiart1ev1aaatCvAUfeBSjuyZL2yd9gzLbvyNv2Caerbhv2BYDwAHbqedmvETj2BSbqee0evGueE0jxyaibaiKI8=vI8tuQ8FMI8Gi=hEeeu0xXdbba9frFj0=OqFfea0dXdd9vqai=hGuQ8kuc9pgc9s8qqaq=dirpe0xb9q8qiLsFr0=vr0=vr0dc8meaabaqaciGacaGaaeqabaqadeqadaaakeaacqaHdpWCdaqhaaWcbaGaamOqaiaadMeacaWGpbaabaGaaGOmaaaaaaa@3842@ was obtained by taking the variance of the gene expression measures from the culture dilution σCUL2
 MathType@MTEF@5@5@+=feaafiart1ev1aaatCvAUfeBSjuyZL2yd9gzLbvyNv2Caerbhv2BYDwAHbqedmvETj2BSbqee0evGueE0jxyaibaiKI8=vI8tuQ8FMI8Gi=hEeeu0xXdbba9frFj0=OqFfea0dXdd9vqai=hGuQ8kuc9pgc9s8qqaq=dirpe0xb9q8qiLsFr0=vr0=vr0dc8meaabaqaciGacaGaaeqabaqadeqadaaakeaacqaHdpWCdaqhaaWcbaGaam4qaiaadwfacaWGmbaabaGaaGOmaaaaaaa@384C@ and subtracting σEXP2
 MathType@MTEF@5@5@+=feaafiart1ev1aaatCvAUfeBSjuyZL2yd9gzLbvyNv2Caerbhv2BYDwAHbqedmvETj2BSbqee0evGueE0jxyaibaiKI8=vI8tuQ8FMI8Gi=hEeeu0xXdbba9frFj0=OqFfea0dXdd9vqai=hGuQ8kuc9pgc9s8qqaq=dirpe0xb9q8qiLsFr0=vr0=vr0dc8meaabaqaciGacaGaaeqabaqadeqadaaakeaacqaHdpWCdaqhaaWcbaGaamyraiaadIfacaWGqbaabaGaaGOmaaaaaaa@3855@, that is:

σBIO2
 MathType@MTEF@5@5@+=feaafiart1ev1aaatCvAUfeBSjuyZL2yd9gzLbvyNv2Caerbhv2BYDwAHbqedmvETj2BSbqee0evGueE0jxyaibaiKI8=vI8tuQ8FMI8Gi=hEeeu0xXdbba9frFj0=OqFfea0dXdd9vqai=hGuQ8kuc9pgc9s8qqaq=dirpe0xb9q8qiLsFr0=vr0=vr0dc8meaabaqaciGacaGaaeqabaqadeqadaaakeaacqaHdpWCdaqhaaWcbaGaamOqaiaadMeacaWGpbaabaGaaGOmaaaaaaa@3842@ = σCUL2
 MathType@MTEF@5@5@+=feaafiart1ev1aaatCvAUfeBSjuyZL2yd9gzLbvyNv2Caerbhv2BYDwAHbqedmvETj2BSbqee0evGueE0jxyaibaiKI8=vI8tuQ8FMI8Gi=hEeeu0xXdbba9frFj0=OqFfea0dXdd9vqai=hGuQ8kuc9pgc9s8qqaq=dirpe0xb9q8qiLsFr0=vr0=vr0dc8meaabaqaciGacaGaaeqabaqadeqadaaakeaacqaHdpWCdaqhaaWcbaGaam4qaiaadwfacaWGmbaabaGaaGOmaaaaaaa@384C@ - σEXP2
 MathType@MTEF@5@5@+=feaafiart1ev1aaatCvAUfeBSjuyZL2yd9gzLbvyNv2Caerbhv2BYDwAHbqedmvETj2BSbqee0evGueE0jxyaibaiKI8=vI8tuQ8FMI8Gi=hEeeu0xXdbba9frFj0=OqFfea0dXdd9vqai=hGuQ8kuc9pgc9s8qqaq=dirpe0xb9q8qiLsFr0=vr0=vr0dc8meaabaqaciGacaGaaeqabaqadeqadaaakeaacqaHdpWCdaqhaaWcbaGaamyraiaadIfacaWGqbaabaGaaGOmaaaaaaa@3855@

**Figure 3:**
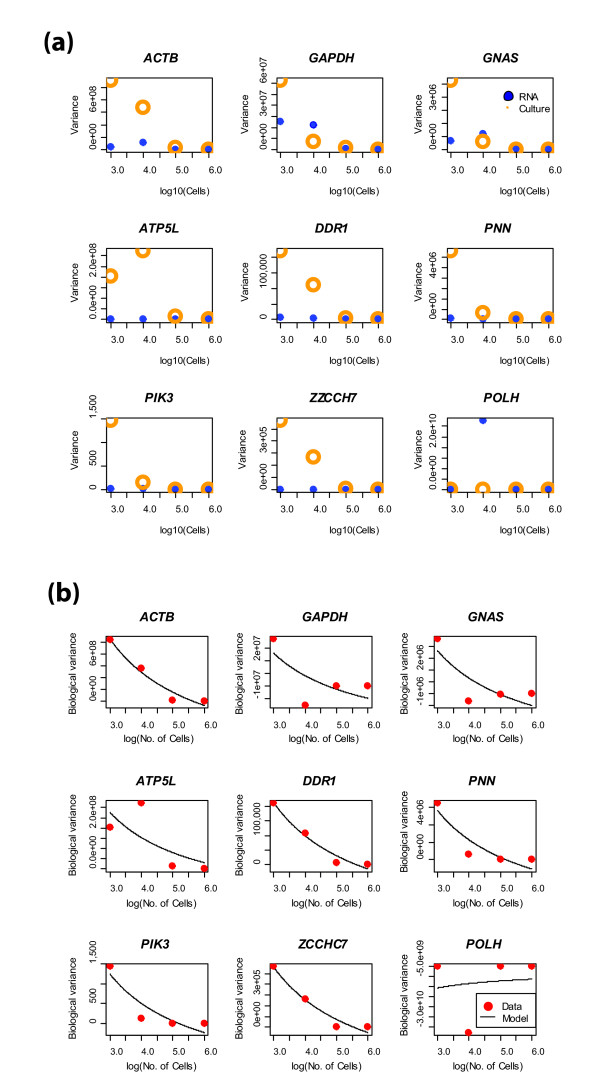
**(a) **Variances calculated from the experimental data for each step of the serial dilution series; variances from the RNA dilution are represented by solid blue circles, variances from the cell culture dilution are represented by the open orange circles. **(b) **Estimates of biological variability obtained from the validation experiment using quant values are shown by red dots; the trend predicted by our analytical model is shown by the bold black line. Data are displayed for nine genes targeted in our validation experiment.

The results, plotted as a function of the number of cells assayed, is shown in Figure 3.

As we assume gene expression is Poisson, with mean *λ*, we can estimate the average expression per cell using simple linear regression, where the estimated biological variability is fit to a function of the form λlog⁡10N+I
 MathType@MTEF@5@5@+=feaafiart1ev1aaatCvAUfeBSjuyZL2yd9gzLbvyNv2Caerbhv2BYDwAHbqedmvETj2BSbqee0evGueE0jxyaibaiKI8=vI8tuQ8FMI8Gi=hEeeu0xXdbba9frFj0=OqFfea0dXdd9vqai=hGuQ8kuc9pgc9s8qqaq=dirpe0xb9q8qiLsFr0=vr0=vr0dc8meaabaqaciGacaGaaeqabaqadeqadaaakeaadaWcaaqaaiabeU7aSbqaaiGacYgacaGGVbGaai4zamaaBaaaleaacaaIXaGaaGimaaqabaGccaWGobaaaiabgUcaRiaadMeaaaa@3BEF@, where *I *represents a linear offset of the biological variability. We can interpret *I *as the value that, along with the estimate of λ, gives the approximate number of cells required in the assay for the biological variability effects to be negligible through the expression:

Nneg=exp⁡(λ|I|)
 MathType@MTEF@5@5@+=feaafiart1ev1aaatCvAUfeBSjuyZL2yd9gzLbvyNv2Caerbhv2BYDwAHbqedmvETj2BSbqee0evGueE0jxyaibaiKI8=vI8tuQ8FMI8Gi=hEeeu0xXdbba9frFj0=OqFfea0dXdd9vqai=hGuQ8kuc9pgc9s8qqaq=dirpe0xb9q8qiLsFr0=vr0=vr0dc8meaabaqaciGacaGaaeqabaqadeqadaaakeaacaWGobWaaSbaaSqaaiaad6gacaWGLbGaam4zaaqabaGccqGH9aqpciGGLbGaaiiEaiaacchadaqadaqaamaalaaabaGaeq4UdWgabaGaaiiFaiaadMeacaGG8baaaaGaayjkaiaawMcaaaaa@40FB@

At a population size of *N*_*neg*_, the stochastic signatures in gene expression are expected to be virtually non-existent. For 8 of 9 genes a good fit to the model is obtained with R^2 ^ranging from 0.68 to 0.98 (Table 2). The remaining gene, *POLH*, had the lowest expression level on the Affymetrix GeneChip™ and in a number of replicate qRT-PCR assays its measured expression level fell outside our detectable range. The poor signal to noise, combined with a smaller number of measurements, easily explain our failure to fit the Poisson model. Nevertheless, for the remaining genes the results provide evidence to support a model of gene expression described by Poisson statistics.

**Figure 4:**
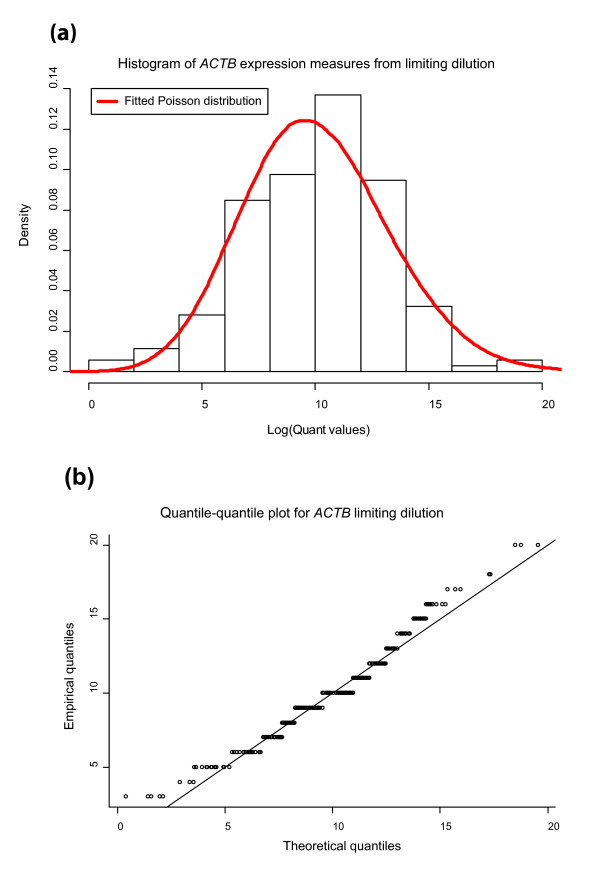
**(a) **Histogram of gene expression values (log(quant values)) of *ACTB *obtained from the limiting dilution experiment. A fit to paired Poisson distributions also suggests that these data represent expression from a single cell rather than one or more cells. **(b) **Quantile-quantile plot.

To further validate this model, we conducted a second experiment in which we assayed *ACTB *gene expression in single cells. We performed a limiting dilution on cultured SW620 cells and measured gene expression using one 384-well qRT-PCR assay plate (360 samples in total) where each well should contain either 0 or 1 cell. Cells were individually lysed in the PCR plate, DNA-ase was added to remove contaminating genomic DNA, and *ACTB *gene expression was measured. The results, shown in Figure 4, indicate that *ACTB *gene expression in single cells follows a Poisson distribution, with a mean quant value of 2,888,388 (or 31.33 cycles). Because we are unable to know with certainty how many cells were present in each well (we assume that this is 0 or 1 but, due to the possibility of imperfect mixing, there is a chance there could be more than one cell per well for a small number of wells), it is possible that an alternative explanation exists. It may be that fixed concentrations of *ACTB *RNA exist in each cell, and as a result our histogram in Figure 4 represents not a distribution of expression but a distribution of cell counts per well instead. To distinguish between these two situations, we fitted a mixture model with two Poisson distributions to the histogram using the expectation-maximization (EM) algorithm [10]. If the histogram represented cell counts, then we would expect the two Poisson distributions to be centred on mean values of X¯
 MathType@MTEF@5@5@+=feaafiart1ev1aaatCvAUfeBSjuyZL2yd9gzLbvyNv2Caerbhv2BYDwAHbqedmvETj2BSbqee0evGueE0jxyaibaiKI8=vI8tuQ8FMI8Gi=hEeeu0xXdbba9frFj0=OqFfea0dXdd9vqai=hGuQ8kuc9pgc9s8qqaq=dirpe0xb9q8qiLsFr0=vr0=vr0dc8meaabaqaciGacaGaaeqabaqadeqadaaakeaaceWGybGbaebaaaa@3422@ and 2X¯
 MathType@MTEF@5@5@+=feaafiart1ev1aaatCvAUfeBSjuyZL2yd9gzLbvyNv2Caerbhv2BYDwAHbqedmvETj2BSbqee0evGueE0jxyaibaiKI8=vI8tuQ8FMI8Gi=hEeeu0xXdbba9frFj0=OqFfea0dXdd9vqai=hGuQ8kuc9pgc9s8qqaq=dirpe0xb9q8qiLsFr0=vr0=vr0dc8meaabaqaciGacaGaaeqabaqadeqadaaakeaaceWGybGbaebaaaa@3422@. Estimates of these parameters were 0.05195 and 10.69 (moreover the relative mixing proportions were 0.0001 and 0.9999), indicating strongly in favor of the first interpretation, that Figure 4 represents a single cell distribution of RNA expression with little, if any, contribution from samples containing multiple cells.

## Conclusion

Although evidence for stochastic processes in biology has been mounting for quite some time, there has only been a single published report of the variability of gene expression in single cells, which did not provide an underlying statistical model for mRNA representation within the cell [1]. While this may seem to be minor, it represents a significant gap in our knowledge if we are to construct the sort of predictive models that are the aim of systems biology.

While we tend to think of a tissue sample as being homogeneous and to discuss levels of gene expression in terms of absolute numbers of copies per cell, our evidence indicates that gene expression levels obey simple and predictable Poisson statistics. When we imagine a gene expressed at 'five copies per cell', there clearly must be a range, with some cells expressing very few or no copies while others express the same gene at high levels and the Poisson distribution specifies the likelihood that any particular number of transcripts will be observed within a population of cells. In support of this proposed model, we provide experimental data that demonstrate precisely the behavior we predict for the variance as a function of the number of cells we sample. The evidence supporting this comes directly from sampling statistics: the variance in gene expression levels decays as 1/*N*, where *N *is the number of cells sampled. The beauty of this result is that it can be measured experimentally even for genes such as *PIK3 *that are expressed at very low levels and that such measurements can be used to estimate commonly quoted properties of the distribution, such as the average expression level. One caveat, of course, is that we are only observing steady state gene expression and have not taken into account the effects of cellular perturbations in which the overall patterns of expression may alter as cells begin transcriptional activity at different times so that the population average at any point may not appear Poisson. However, our results suggest that when 'bursts' of transcription (or translation) do occur, one must consider the probability distribution reflecting the number of molecules produced.

We also demonstrate something subtle but important: the effects of stochastic events occurring at a cellular level can be observed by looking at small but experimentally accessible numbers of cells. This suggests that other stochastic events occurring in single cells, even complex interactions in pathways, may reveal themselves through the analysis of samples of mesoscopic size. In many ways, this situation is analogous to one in statistical mechanics and thermodynamics. While we understand that the Ideal Gas Law describes gas dynamics for macroscopic samples, we know that, on a microscopic scale, the behavior of the gas molecules themselves are described by the Maxwell-Boltzman distribution. But observing individual molecules is essentially impossible. The compromise is to look at small numbers of molecules - mesoscopic samples - where one can begin to see deviations from the ideal gas behavior. Our hope in presenting this work is to open the door to a new approach to the study of biological systems in which, working with small but tractable numbers of cells, we can begin to explore the stochastic components of cellular processes. Understanding these effects will be essential if we are to develop useful systems biology approaches that do more than model average behavior but instead provide insight into the processes that lead away from the average to the development of disease phenotypes.

## Materials and methods

### SW620 cell culture

Cells from the human colon cancer cell line SW620 (American Type Culture Collection) were seeded in 100 mm tissue culture dishes using Dulbecco's Modified Eagle's Medium supplemented with 10% fetal bovine serum and 1% penicillin/streptomycin. Cells were cultured to a confluence of 1.0 × 10^7 ^cells at 37°C and 5% CO_2_.

### RNA extraction

RNA was extracted and purified using the Versagene RNA Purification Kit (Gentra Systems, Minneapolis, MN, USA) and the Absolutely RNA Miniprep and Microprep kits (Stratagene, La Jolla, CA, USA) according to each manufacturer's instructions. After RNA extraction from 1 × 10^7 ^cells using the Versagene RNA Purification kit, the RNA was subjected to a series of 4 1:10 dilutions to a final dilution of 1 × 10^3 ^cells, with 9 replicates at each RNA dilution level. With another tissue culture dish containing 1 × 10^7 ^cells, cells were removed from the monolayer and subjected to the same 1:10 dilution series prior to RNA extraction. After 4 dilutions, a final dilution of 1 × 10^3 ^cells was achieved, with 9 replicates at each cell dilution level. RNA was then extracted from each replicate in the dilution series using the Absolutely RNA Miniprep and Microprep kits.

### Affymetrix microarray analysis

RNA from SW620 cells was prepared, labeled, and hybridized in triplicate to the Affymetrix U133Plus2 GeneChip™ according to the manufacturer's instructions (Affymetrix, Santa Clara, CA, USA). Probe sets were retained only if they appeared in three replicate arrays; the retained probe sets were assigned expression measures using the robust multi-array statistic developed by Irizarry *et al*. [11]. Probe sets were matched using HUGO gene symbols. Genes were then sorted by expression values into low, medium and high expression groups based on quartiles (the lowest quartile was discarded). We selected candidate genes from these three groups based on information found in the literature. RT-PCR was performed on these genes to determine their expression levels, relative to each other. The final nine genes were selected to represent a reasonable degree of coverage across these three levels.

### RT-PCR

Total RNA was extracted from cells according to the procedures described above. These RNA samples were then reverse transcribed to produce cDNA using reagents from the TaqMan reverse transcription kit (Applied Biosystems, Foster City, CA, USA) and then subjected to quantitative PCR using SYBR Green (Applied Biosystems). SYBR Green incorporation was detected in real time using the ABI Prism 7900HT system and expression was quantified using 18S ribosomal RNA (Ambion, Austin, TX, USA) as a standard curve for normalization. Forward and reverse primer pair sequences (Invitrogen, Carlsbad, CA, USA) used for RT-PCR were: ACTB, (GGACTTCGAGCAAGAGATGG, AGGAAGGAAGGCTGGAAGAG); ATP5L, (CAAGGTTGAGCTGGTTCCTC, CACCAAACCATTCAGCACAG); GAPDH, (GAGTCAACGGATTTGGTCGT, GATCTCGCTCCTGGAAGATG); GNAS, (TGAACGTGCCTGACTTTGAC, TCCACCTGGAACTTGGTCTC); DDR1, (AATGAGGACCCTGAGGGAGT, CCGTCATAGGTGGAGTCGTT); PIK3, (GAGGAGGTGCTGTGGAATGT, GAGGAGGTGCTGTGGAATGT); PNN, (AGCGCACACGTAGAGACCTT, CCGCTTTTGCCTTTCAGTAG); POLH, (ATGGGACCGTAACTCAGCAC, TCAGGCTTGCCTGTAGGATT); ZCCHC7, (GGACCCAGCGGTACTATTCA, GGCTGGACAGGAATACAGGA).

### Single cell RT-PCR

SW620 human colon cancer cells were cultured according to the procedures described above and harvested at a confluence of 2.41 × 10^7 ^cells. Cells were then diluted in sterile water to a final concentration of 1 cell/μl. A 96-well plate, each well containing one cell, was placed in a thermal cycler at 95°C for two minutes to pop the cells. DNase I was added to degrade DNA at 37°C for 1 hour. EDTA was added at a final concentration of 5 mM to protect the RNA, then incubated at 75°C for 10 minutes to deactivate the DNase I. Resulting RNA from single cells was then subjected to RT-PCR according to the procedures described above. One 384-well plate was used, yielding 360 samples in total (remaining wells were devoted to obtaining measurements for standard curves and negative controls).

### Regression modeling

Figure 4 represents curves fitted using simple linear regression modeling of the empirical data. The covariate in the regression model *N *(representing the number of cells) has been log_10_-transformed.

Based on derivations from the theoretical model, we expect to see the empirical variances, as calculated from our experimental data, to behave according to λN
 MathType@MTEF@5@5@+=feaafiart1ev1aaatCvAUfeBSjuyZL2yd9gzLbvyNv2Caerbhv2BYDwAHbqedmvETj2BSbqee0evGueE0jxyaibaiKI8=vI8tuQ8FMI8Gi=hEeeu0xXdbba9frFj0=OqFfea0dXdd9vqai=hGuQ8kuc9pgc9s8qqaq=dirpe0xb9q8qiLsFr0=vr0=vr0dc8meaabaqaciGacaGaaeqabaqadeqadaaakeaadaWcaaqaaiabeU7aSbqaaiaad6eaaaaaaa@35C4@, in other words, a decay following a 1N
 MathType@MTEF@5@5@+=feaafiart1ev1aaatCvAUfeBSjuyZL2yd9gzLbvyNv2Caerbhv2BYDwAHbqedmvETj2BSbqee0evGueE0jxyaibaiKI8=vI8tuQ8FMI8Gi=hEeeu0xXdbba9frFj0=OqFfea0dXdd9vqai=hGuQ8kuc9pgc9s8qqaq=dirpe0xb9q8qiLsFr0=vr0=vr0dc8meaabaqaciGacaGaaeqabaqadeqadaaakeaadaWcaaqaaiaaigdaaeaacaWGobaaaaaa@34CB@ relationship with some scaling factor λ involved. To estimate this scaling factor we fitted a simple linear regression, using the transformed covariate 1/N* (where N* = log_10_*N*). We did not force the regression line to pass through the origin, and hence allowed for a non-zero intercept in our model, which we denote as *I*. To derive a reasonable interpretation for the intercept *I*, imagine that as the variance approaches zero:

I=−λlog⁡N
 MathType@MTEF@5@5@+=feaafiart1ev1aaatCvAUfeBSjuyZL2yd9gzLbvyNv2Caerbhv2BYDwAHbqedmvETj2BSbqee0evGueE0jxyaibaiKI8=vI8tuQ8FMI8Gi=hEeeu0xXdbba9frFj0=OqFfea0dXdd9vqai=hGuQ8kuc9pgc9s8qqaq=dirpe0xb9q8qiLsFr0=vr0=vr0dc8meaabaqaciGacaGaaeqabaqadeqadaaakeaacaWGjbGaeyypa0JaeyOeI0YaaSaaaeaacqaH7oaBaeaaciGGSbGaai4BaiaacEgacaWGobaaaaaa@3B55@

An easier way to interpret this is with respect to *N*, and if we rearrange the previous equation we get:

N=exp⁡(−λI)
 MathType@MTEF@5@5@+=feaafiart1ev1aaatCvAUfeBSjuyZL2yd9gzLbvyNv2Caerbhv2BYDwAHbqedmvETj2BSbqee0evGueE0jxyaibaiKI8=vI8tuQ8FMI8Gi=hEeeu0xXdbba9frFj0=OqFfea0dXdd9vqai=hGuQ8kuc9pgc9s8qqaq=dirpe0xb9q8qiLsFr0=vr0=vr0dc8meaabaqaciGacaGaaeqabaqadeqadaaakeaacaWGobGaeyypa0JaciyzaiaacIhacaGGWbWaaeWaaeaacqGHsisldaWcaaqaaiabeU7aSbqaaiaadMeaaaaacaGLOaGaayzkaaaaaa@3CE9@

and, since this relationship only holds for values of *N *when the variance approaches zero or negligible levels, we denote this equation as:

Nneg=exp⁡(−λI)
 MathType@MTEF@5@5@+=feaafiart1ev1aaatCvAUfeBSjuyZL2yd9gzLbvyNv2Caerbhv2BYDwAHbqedmvETj2BSbqee0evGueE0jxyaibaiKI8=vI8tuQ8FMI8Gi=hEeeu0xXdbba9frFj0=OqFfea0dXdd9vqai=hGuQ8kuc9pgc9s8qqaq=dirpe0xb9q8qiLsFr0=vr0=vr0dc8meaabaqaciGacaGaaeqabaqadeqadaaakeaacaWGobWaaSbaaSqaaiaad6gacaWGLbGaam4zaaqabaGccqGH9aqpciGGLbGaaiiEaiaacchadaqadaqaaiabgkHiTmaalaaabaGaeq4UdWgabaGaamysaaaaaiaawIcacaGLPaaaaaa@3FE8@

to distinguish from all other values of *N*.

### Poisson distribution analysis

Empirical evidence in support of the assumption that gene expression levels follow a Poisson distribution was strengthened by two simple statistical analyses. First, a histogram (Figure 4) of the gene expression levels obtained from the limiting dilution experiment for *ACTB *resembles the expected probability distribution function (values are skewed to the left). Second, we constructed a quantile-quantile plot, comparing empirical quantiles based on the *ACTB *gene expression levels with theoretical quantiles expected for a Poisson distribution (with mean equal to the observed mean). Quantiles, like percentiles and quartiles, represent summary statistics of the data that help us gauge the spread of the distribution of data points. For instance, the 25th percentile represents the value that 25% of the lowest data points fall below. While percentiles are achieved by dividing the data into 100 sections, and quartiles represent divisions into 4, a quantile represents a generalized term for any division. Quartiles and percentiles are actually 4-quantiles and 100-quantiles, respectively. The idea behind the quantile-quantile plot is to compare how the data points are distributed (relative to each other) in the empirical sample (where the distribution is typically unknown) with a theoretical sample that has been simulated under a distributional assumption.

The majority of the data follows the Poisson assumption; some apparent deviation was likely to be a result of experimental artefacts. A two-component Poisson mixture model was fitted to the histogram of RT-PCR quant values using a quasi-Newton method with constraints (via the optim function in R). The algorithm was terminated when the relative difference in the log-likelihood functions was less than 1.4901 × 10^-8^.

### Data and software availability

All data generated and analyzed in this manuscript as well as the R code used in the analysis and a tutorial outlining the various steps are available from [12] so that readers can reproduce our results and apply a similar analysis to their own datasets.

## Additional data file

The following additional data are available with the online version of this paper. Additional data file 1 is a .zip file containing the qRT-PCR data analyzed in this manuscript, the software (as R code) used to perform the analysis and produce the figures presented, and instructions on how to install R and perform the analysis as well as a "README" that explicitly describes each file in the .zip archive.

## Supplementary Material

Additional data file 1 AZIP file containing the qRT-PCR data analyzed in this manuscript, the software (as R code) used to perform the analysis and produce the figures presented, and instructions on how to install R and perform the analysis as well as a "README" that explicitly describes each file in the .zip archive. Click here for file

## Figures and Tables

**Table 1:** Genes featured in the validation experiment

Low	Medium	High
*PIK3*	*ATP5L*	*ACTB*
*ZCCH7*	*PNN*	*GAPDH*
*POLH*	*DDR1*	*GNAS*

Genes that featured in the validation experiment were selected based on demonstrated levels of 'high', 'medium' and 'low' expression.

**Table 2:** Estimates of model parameters λ and *I*

Gene	Correlation	λ estimate	*I *(intercept estimate)
*ACTB*	0.9818035	6.802453 × 10^9^	-1.208535 × 10^9^
*GAPDH*	0.6946698	2.122443 × 10^8^	-4.484740 × 10^7^
*DDR1*	0.9838246	1.370801 × 10^6^	-2.441642 × 10^5^
*PIK3*	0.9148329	8.885468 × 10^3^	-1.719060 × 10^3^
*PNN*	0.9160348	4.000586 × 10^7^	-7.723061 × 10^6^
*ZCCHC7*	0.9827101	3.656176 × 10^6^	-6.591370 × 10^5^
*POLH*	0.1149602	-2.590916 × 10^10^	-2.015157 × 10^9^
*ATP5L*	0.6793007	1.464513 × 10^9^	-2.127757 × 10^8^
*GNAS*	0.8224466	2.762874 × 10^7^	-5.596301 × 10^6^

λ and *I *were estimated by regressing biological variability on λlog⁡10N+I
 MathType@MTEF@5@5@+=feaafiart1ev1aaatCvAUfeBSjuyZL2yd9gzLbvyNv2Caerbhv2BYDwAHbqedmvETj2BSbqee0evGueE0jxyaibaiKI8=vI8tuQ8FMI8Gi=hEeeu0xXdbba9frFj0=OqFfea0dXdd9vqai=hGuQ8kuc9pgc9s8qqaq=dirpe0xb9q8qiLsFr0=vr0=vr0dc8meaabaqaciGacaGaaeqabaqadeqadaaakeaadaWcaaqaaiabeU7aSbqaaiGacYgacaGGVbGaai4zamaaBaaaleaacaaIXaGaaGimaaqabaGccaWGobaaaiabgUcaRiaadMeaaaa@3BEF@. We also computed the Pearson correlation coefficients to measure the correlation between the biological variability estimates from our analytical model and the biological variability observed in the validation experiment.
