# Supplementary material for: Inferring steady state single-cell gene expression distributions from analysis of mesoscopic samples
Source: Genome Biol. 2006 Dec 14;7(12):R119. doi: 10.1186/gb-2006-7-12-r119 (PMC1794432; doi:10.1186/gb-2006-7-12-r119)
Supplement: Additional data file 1 — AZIP file containing the qRT-PCR data analyzed in this manuscript, the software (as R code) used to perform the analysis and produce the figures presented, and instructions on how to install R and perform the analysis as well as a "README" that explicitly describes each file in the .zip archive. [file gb-2006-7-12-r119-S1.zip › stochastic/Supplementary Info.pdf]

Supplementary Information to "Inferring steady state single-cell gene expression distributions from analysis of mesoscopic samples"
by Jessica C. Mar, Renee Rubio and John Quackenbush

We have made available all our experimental data and the code used in the data analysis in the form of a zip file `stochastic.zip`. Please unzip the zip file and consult the README.txt for brief instructions on how to run the code. For a more detailed explanation of our data analysis, follow the instructions outlined in this pdf.

First you need to have access to a working version of R (we used the Windows version 2.2.1 but the code is supported by any version). This software can be downloaded (via the appropriate mirror site) from:

http://cran.r-project.org/mirrors.html

The first file to execute is `func.R`. This file contains functions that are later called upon in steps of our data analysis and also reads in the raw data and formats it in R objects that we can then easily use to plot and analyze.

When you start your R session, it's important that you are in the correct working directory. Cut and paste the following command (omitting the > character) into the R session window, replacing `path` with the path address that corresponds to the location that the zip file was extracted to (in other words the location of the README.txt file).

```
> setwd("path")
```

To execute a file in R, cut and paste the following command into the R session window. Note that throughout this guide, we will use `courier` to denote executable R code, and that code can be executed by cutting and pasting the code into the R session window. We will use a > character to denote the start of any R code (omit the > character when cutting and pasting over to R).

```
> source("Rcode/func.R")
```

Our qRT-PCR experiments were designed to fit onto 6 384-well plates (3 for the cell culture serial dilutions, 3 for the RNA serial dilutions). Our raw data came in the form of 6 ASCII-text files which are stored under the folder labeled `Serial_Dilution_Data`:

Culture Dilution Plate 1 Results.txt	RNA Dilution Plate 1 Results.txt
Culture Dilution Plate 2 Results.txt	RNA Dilution Plate 2 Results.txt
Culture Dilution Plate 3 Results.txt	RNA Dilution Plate 3 Results.txt

In the following paragraphs, we pull out important parts of the code from the `func.R` file to illustrate the steps involved in formatting our data. Note, by executing the `func.R` file, you would already have generated all the steps necessary so you do not actually need

to replicate them by pasting the following code chunks into the R session but may do so to further understand the steps we have performed.

```
> rnaplate1 <- extractPCRData("RNA Dilution Plate 1
Results.txt", nRow=356)
```

The function `extractPCRData` takes the raw data file, removes extraneous information (e.g. experimental information reported by the machine) and summarizes the data by taking means over values from replicated wells.

The next block of code reformats all raw data so that we have a single text file for each gene with data from both cell culture and RNA serial dilutions. The code generates these text files and stores them in the folder labeled `Serial_Dilution_Data`.

```
> rna.allplates <- rbind(rnaplate1, rnaplate2, rnaplate3) ;
   cul.allplates <- rbind(culplate1, culplate2, culplate3)
  genes <- c("ACTB", "GAPDH", "GNAS", "ATP5L", "DDR1",
     "PNN", "PIK3", "ZCCHC7", "POLH")
  dil.steps <- c("10^3", "10^4", "10^5", "10^6")

 for( i in 1:length(genes) ){
     rna.xx <- rna.allplates[grep(genes[i],
     rownames(rna.allplates)),]
     rna.fileName <- paste(genes[i], "_RNA_raw.txt",
     sep="")
     rna.xx[,c(2,4,6)] <- log(rna.xx[,c(2,4,6)], 10)
     write.table(rna.xx, rna.fileName, sep="\t", quote=F)
     cul.xx <- cul.allplates[grep(genes[i],
     rownames(cul.allplates)),]
     cul.fileName <- paste(genes[i], "_CUL_raw.txt",
     sep="")
     cul.xx[,c(2,4,6)] <- log(cul.xx[,c(2,4,6)], 10)
     write.table(cul.xx, cul.fileName, sep="\t", quote=F)
}
```

The function `readGeneInfo` takes the text file for a particular gene (like the ones created in the last step), and computes the variance and mean calculations across the replicated measures for each dilution step.

The function `calcVarComp` computes the biological variance estimates for a particular gene across the dilution steps in the serial dilution.

The function `calcExpVarComp` computes estimates of technical variability for a particular gene (we will use this data in Figure 3A).

```
> actb.dat <- readGeneInfo("ACTB", readRaw=T, dil.steps)
```

```
bio.actb <- calcVarComp(actb.dat, ct=F)
err.actb <- calcExpVarComp(actb.dat, ct=F)
```

Next, we will perform the data analyses and produce Figures 1A, 1B, 3A and 3B. The code to do this is contained in the file `analysis.R`. To execute the code stored in this file, cut and paste the following command into the R session window (omitting the `>` character).

```
> source("Rcode/analysis.R")
```

In the next few paragraphs we'll explain which individual parts of the code from this file were used to produce each separate figure from the paper.

We used the function `genCellExp.poi` to generate the simulated data for the virtual cell populations under the Poisson assumptions. The function takes three input arguments. The `lam` argument denotes the intensity parameter for the Poisson distribution that will be simulated; the `R` argument denotes the number of replicates to perform (in Figure 1A, `R = 1000`, in Figure 1B, `R = 10`) and the `Nvals` argument represents the vector of population sizes to simulate data for.

```
> genCellExp.poi <- function(lam, R, Nvals){

    res.mat <- matrix(0, nrow=R, ncol=length(Nvals) )
    for( n in 1:length(Nvals) ){
        res.mean <- NULL
        for( r in 1:R ){
            samp <- rpois(Nvals[n], lam)
            res.mean <- c(res.mean, mean(samp))
        }
        res.mat[,n] <- res.mean
    }

   mean.val <- apply(res.mat, 2, mean)
   std.val <- apply(res.mat, 2, sd)
   upp.val <- mean.val + std.val
   low.val <- mean.val - std.val

    x <- list(res.mat=res.mat, mean.val=mean.val,
     std.val=std.val, upp.val=upp.val, low.val=low.val)
    return(x)
}
```

For Figure 1A, we used `R = 1000` and generated data for five different values of λ. Since this step is fairly computationally intense, it can take a considerable amount of time for R to generate all the simulations. Therefore we have made our simulation data available in the folder `Simulated_Data` (note: if you prefer to simulate your own

data, the code to do this is also in `analysis.R` but has been commented out with the # character). The folder Simulated_Data contains two subfolders: `1000Replicates` which stores the data simulated under `R = 1000` (Figure 1A), and `10Replicates` which stores the data simulated under `R = 10` (Figure 1B). The data are stored as R objects; you can load these objects using the `load` command. As an example, you can cut and paste the following command in the R session window to load the simulated data for $\lambda = 0.5$.

```
> load("./Simulation_Data/1000Replicates/lowres.Rdata")
```

Alternatively here is the code one would use to generate simulated data for $\lambda = 0.5$:

```
> R <- 1000 ; Nvals <- seq(from=500, to=5000, by=5)
  low.lam <- .5
  low.res <- genCellExp.poi(low.lam, R, Nvals)
```

The code used to produce Figure 1A was:

```
> y.rang <- range(c(low.res$std.val/low.res$mean.val,
     mid.res$std.val/mid.res$mean.val,
     hig.res$std.val/hig.res$mean.val,
     xhig.res$std.val/xhig.res$mean.val,
     xxhig.res$std.val/xxhig.res$mean.val))

  Nvals <- seq(from=500, to=5000, by=5)
  win.graph(22, 12) # note for non-windows, replace this
                    # with x11()
  plot(Nvals, low.res$std.val/low.res$mean.val,
     ylim=y.rang, ylab="Standardized Standard Deviation",
     xlab="Number of Cells (N)", pch=20)
     points(Nvals, mid.res$std.val/mid.res$mean.val,
     col="red", pch=20)
     points(Nvals, hig.res$std.val/hig.res$mean.val,
     col="blue", pch=20)
     points(Nvals, xhig.res$std.val/xhig.res$mean.val,
     col="green", pch=20)
     points(Nvals, xxhig.res$std.val/xxhig.res$mean.val,
     col="orange", pch=20)
     legend(3910, .063, c("LOW LAMBDA (0.5)", "MID LAMBDA
     (5)", "HIGH LAMBDA (50)", "HIGHER LAMBDA (500)",
     "HIGHEST LAMBDA (5000)"),
     col=c("black", "red", "blue", "green", "orange"),
     pch=rep(20,5), bty="n")
     text(1200, .059, c("1000 replicates"), font=4)
```

For Figure 1B, we dropped the number of replicates from 1000 to `R = 10` and simulated data for the same five values of λ that were used for the simulations shown in Figure 1A.

```
> R <- 10
  low.res.sd <- genCellExp.poi(low.lam, R, Nvals)
```

The code used to produce Figure 1B is therefore very similar to the code used for Figure 1A. One addition though is the super-position of the analytical solution.

```
> Nvals <- seq(from=500, to=5000,
     length=length(low.res.sd$std.val))
  Nvals.smooth <- seq(from=500, to=5000, length=1000)

 win.graph(22, 12)
 plot(Nvals, low.res.sd$std.val/low.res.sd$mean.val,
     ylim=y.rang, ylab="Standardized Standard Deviation",
     xlab="Number of Cells (N)", pch=20)
     points(Nvals, mid.res.sd$std.val/mid.res.sd$mean.val,
     col="red", pch=20)
     points(Nvals, hig.res.sd$std.val/hig.res.sd$mean.val,
     col="blue", pch=20)
     points(Nvals,
     xhig.res.sd$std.val/xhig.res.sd$mean.val, col="green",
     pch=20)
     points(Nvals,
     xxhig.res.sd$std.val/xxhig.res.sd$mean.val,
     col="orange", pch=20)
     lines(Nvals.smooth,
     sqrt(low.lam/Nvals.smooth)/low.lam, col="black",
     lwd=3)
     lines(Nvals.smooth,
     sqrt(mid.lam/Nvals.smooth)/mid.lam, col="red", lwd=3)
     lines(Nvals.smooth,
     sqrt(hig.lam/Nvals.smooth)/hig.lam, col="blue", lwd=3)
     lines(Nvals.smooth,
     sqrt(xhig.lam/Nvals.smooth)/xhig.lam, col="green",
     lwd=3)
     lines(Nvals.smooth,
     sqrt(xxhig.lam/Nvals.smooth)/xxhig.lam, col="orange",
     lwd=3)
     legend(3690, .085, c("LOW LAMBDA (0.5)", "MID LAMBDA
     (5)", "HIGH LAMBDA (50)", "HIGHER LAMBDA (500)",
     "HIGHEST LAMBDA (5000)"),
    col=c("black", "red", "blue", "green", "orange"),
     lty=rep(1,5), pch=rep(20,5), lwd=rep(3,3), bty="n")
     text(800, .085, c("10 replicates"), font=4)
     legend(2000, .085, c("Predicted Result", "Simulated
```

```
     Result"), lty=c(1,0), lwd=c(3,0), col=c("black",
     "black"), pch=c(-1,1), bty="n")
```

Figure 3A is simply a plot of the variances from the two serial dilutions for each of the nine genes.

```
> par(mfrow=c(3,3), pch=20)
  plot(3:6, err.actb, ylim=range(c(err.actb, cul.actb)),
     cex=3, col="blue", xlab="log10(Cells)",
     ylab="Variance", main="ACTB")
     points(3:6, cul.actb, col="orange", pch=1, cex=3,
     lwd=4)
 plot(3:6, err.gapdh, ylim=range(c(err.gapdh, cul.gapdh)),
     cex=3, col="blue", xlab="log10(Cells)",
     ylab="Variance", main="GAPDH")
     points(3:6, cul.gapdh, col="orange", pch=1, cex=3,
     lwd=4)
 plot(3:6, err.gnas, ylim=range(c(err.gnas, cul.gnas)),
     cex=3, col="blue", xlab="log10(Cells)",
     ylab="Variance", main="GNAS")
     points(3:6, cul.gnas, col="orange", pch=1, cex=3,
     lwd=4)
     legend(4.5, 4805925, c("RNA", "Culture"),
     col=c("blue", "orange"), pch=c(20,20))
 plot(3:6, err.atp, ylim=range(c(err.atp, cul.atp)), cex=3,
     col="blue", xlab="log10(Cells)", ylab="Variance",
     main="ATP5L")
     points(3:6, cul.atp, col="orange", pch=1, cex=3,
     lwd=4)
 plot(3:6, err.ddr, ylim=range(c(err.ddr, cul.ddr)), cex=3,
     col="blue", xlab="log10(Cells)", ylab="Variance",
     main="DDR1")
     points(3:6, cul.ddr, col="orange", pch=1, cex=3,
     lwd=4)
 plot(3:6, err.pnn, ylim=range(c(err.pnn, cul.pnn)), cex=3,
     col="blue", xlab="log10(Cells)", ylab="Variance",
     main="PNN")
     points(3:6, cul.pnn, col="orange", pch=1, cex=3,
     lwd=4)
 plot(3:6, err.pik, ylim=range(c(err.pik, cul.pik)), cex=3,
     col="blue", xlab="log10(Cells)", ylab="Variance",
     main="PIK3")
     points(3:6, cul.pik, col="orange", pch=1, cex=3,
     lwd=4)
 plot(3:6, err.zcc, ylim=range(c(err.zcc, cul.zcc)), cex=3,
     col="blue", xlab="log10(Cells)", ylab="Variance", ,
     main="ZZCCH7")
```

```
     points(3:6, cul.zcc, col="orange", pch=1, cex=3,
     lwd=4)
 plot(3:6, err.pol, ylim=range(c(err.pol, cul.pol)), cex=3,
     col="blue", xlab="log10(Cells)", ylab="Variance",
     main="POLH")
     points(3:6, cul.pol, col="orange", pch=1, cex=3,
     lwd=4)
```

For Figure 3B we plotted both estimates of biological variability, and fitted predictions from a regression model. We used the `lm` function to fit the regression models, where the response was `bio.obj`, the R object which stores the biological variance estimates and the covariate was the inverse log10 transformed number of cells. We used a `for` loop to fit this model to each of the nine genes separately.

```
> short.genes <- c("actb", "gapdh", "gnas", "atp", "ddr",
     "pnn", "pik", "zcc", "pol")
  title.genes <- c("ACTB", "GAPDH", "GNAS", "ATP5L",
     "DDR1", "PNN", "PIK3", "ZCCHC7", "POLH")
  inv.logN <- 1/(3:6) ; fit.logN <- seq(from=3, to=6,
     by=.01)

  for( i in 1:length(short.genes) ){
     bio.obj <- get(paste("bio.", short.genes[i], sep=""))
     est.reg <- lm(bio.obj ~ inv.logN)
     est.lambda <- coefficients(est.reg)[[2]]
     est.shift <- coefficients(est.reg)[[1]]
     fit.bio <- est.lambda/fit.logN + est.shift
     assign(paste("bio.fit.", short.genes[i], sep=""),
          fit.bio)
     assign(paste("bio.reg.", short.genes[i], sep=""),
          est.reg)
}
```

The estimates from the regression models were displayed in Table 2. To reproduce a table of these values in R, cut and paste the following code into the R session window:

```
> all.lambda <- NULL ; all.offset <- NULL
 for( i in 1:length(short.genes) ){
     lm.obj <- get(paste("bio.reg.", short.genes[i],
     sep=""))
     all.lambda <- c(all.lambda, coefficients(lm.obj)[[2]])
     all.offset <- c(all.offset, coefficients(lm.obj)[[1]])
 }

 est.mat <- cbind(all.lambda, all.offset)
 row.names(est.mat) <- short.genes
```

The following code computes the Pearson correlation coefficients that also appear in Table 2.

```
> obs.logN <- 3:6
 pred.mat <- matrix(0, ncol=length(obs.logN),
     nrow=length(short.genes))
     row.names(pred.mat) <- short.genes

 for( i in 1:length(short.genes) ){
     j <- (1:length(short.genes))[row.names(est.mat) %in%
     short.genes[i]]
     reg.est <- est.mat[j,]
     pred.mat[j,] <- reg.est[1]/obs.logN + reg.est[2]
 }

 cor.res <- NULL
 for( i in 1:length(short.genes) ){
     bio.exp <- get(paste("bio.", short.genes[i], sep=""))
     cor.res <- c(cor.res, cor(bio.exp, pred.mat[i,],
     method="pearson"))
 }

 names(cor.res) <- short.genes
```

To generate Figure 3B, we used the following code:

```
> win.graph(22,22)
 par(mfrow=c(3,3))
 for( i in 1:length(short.genes) ){
     bio.fit <- get(paste("bio.fit.", short.genes[i],
     sep=""))
     bio.obj <- get(paste("bio.", short.genes[i], sep=""))
     rg <- range(c(bio.fit, bio.obj))
     plot(fit.logN, bio.fit, pch=24, ylim=rg,
     main=paste(title.genes[i]), ylab="Biological
     Variance", xlab="log(No. of Cells)", type="l")
     points(3:6, bio.obj, pch=20, col="red", cex=3)
 }

 legend(4.3, -15e9, c("Data", "Model"), lty=c(-1,1),
     pch=c(20,-1), col=c("red", "black"), cex=1)
```

The remaining figures in the paper consider the fit of the Poisson distribution to the single cell gene expression measures. The code used for this analysis is contained in the file `fitpoisson.R`. To reproduce Figures 4A and 4B, cut and paste the following command into the R session window.

```
> source(“Rcode/fitpoisson.R”)
```

The raw data from the limiting dilution on β-actin (ACTB) is stored in the comma separated file `singleCellData.csv` (this is stored in the folder named `Limiting_Dilution_Data`).

The following paragraphs detail the data analysis steps involved in analyzing this data and producing Figures 4A and 4B. The raw data was read into R and only the Quant values retained (the software outputs both Ct and Quant values). For wells that the software was unable to assign a measure to, their corresponding values were recoded as `NA`.

```
> raw.dat <- read.csv("singleCellData.csv", head=T)
 raw.quant <- raw.dat[,4]
 raw.quant[raw.quant == "Undetermined"] <- NA
 raw.quant <- as.numeric(data.matrix(raw.quant))
```

To get estimates of mRNA copy from the qRT-PCR data we log transformed the Quant values (as a result, 4 wells had negative transformed values, these were discarded).

```
> plquant <- log(raw.quant)[log(raw.quant) > 0]
     # log quant values, keep those that are positive
     # we end up discarding 4
```

In order to construct a quantile-quantile plot we needed to first generate a vector of quantiles based on a simulated distribution.

```
> plquant.t <- rpois(length(plquant), mean(plquant))
     # theoretical quantiles
```

The following code was used to produce Figure 4A:

```
> win.graph(22,16)
 plot(sort(plquant), sort(plquant.t), xlab="Theoretical
     Quantiles", ylab="Empirical   Quantiles",
     main="Quantile-Quantile Plot for ACTB Limiting
     Dilution")
 abline(0,1)
```

For Figure 4B, we constructed a histogram of the ACTB expression measures, and superimposed a density curve in red. This density was estimated from 10^5 independent, identically distributed Poisson random variables generated with intensity parameter equal to the sample mean.

```
> hist(plquant, prob=T, breaks=10, xlab="Log(Quant
     Values)", main="Histogram of  ACTB Expression Measures
     from Limiting Dilution")
```

```
d <- density(rpois(100000, mean(plquant)), bw=.6)
lines(d$x, d$y, lwd=3, col="red")
legend(0, 0.14, c("Fitted Poisson Distribution"),
    col="red", lwd=3)
```

Finally, we fitted a mixture of two Poisson distributions to the distribution of ACTB expression measures to determine if our interpretation of the data was valid, and not corrupted by experimental error.

If we assume that two distinct intensity parameters λ_1 and λ_2 define two separate Poisson distributions, the log-likelihood function for N mRNA copy numbers observed is:

$$\log L(\Psi) = \sum_{i=1}^{N} \log\left(\pi\left\{\frac{e^{-\lambda_1}\lambda_1^{z_i}}{z_i!}\right\} + (1-\pi)\left\{\frac{e^{-\lambda_2}\lambda_2^{z_i}}{z_i!}\right\}\right)$$

where π represents the proportion of copy numbers that belong to the first Poisson distribution (with intensity parameter λ_1) . The unknown parameters are π, λ_1 and λ_2. We have chosen to estimate these parameters using a quasi-Newton method with the `optim` function in R. Since these parameters have natural boundaries ($0 < \pi < 1$; $0 < \lambda_1 < \lambda_2 < max(z_1, \ldots, z_N)$) we used the `L-BFGS-B` option which allowed us to estimate these parameters subject to the imposed constraints.

We coded the log-likelihood function as a separate function (this is required by the `optim` function) where its input arguments were the vector of unknown parameters and the observed counts.

```
> calc.lloghood.auto <- function(theta, yvec){
    pi <- theta[1] ; lambda <- theta[2:3]
    pi <- c(pi, 1-pi)
    llval <- 0
    for( i in 1:length(yvec) ){
         fd <- pi[1]*exp(-
    lambda[1])*lambda[1]^yvec[i]/factorial(yvec[i]) +
    pi[2]*exp(-
    lambda[2])*lambda[2]^yvec[i]/factorial(yvec[i])
    llval <- llval + log(fd)
    }
    return(-llval) # optim minimizes function
}
```

Our unknown parameters were initialized and vectors of upper and lower values for these parameters were created. We used default error tolerances (relative error of 1.4901×10^{-8}).

```
> theta0 <- c(.05, 1, 11)     # init parameters
```

```
theta_l <- c(0.0001,0.0001,0.0001) ; theta_u <- c(.9999,
    max(plquant), max(plquant))

a <- hist(plquant, breaks=length(plquant))
optim(theta0, calc.lloghood.auto, gr=NULL, method="L-BFGS-
    B", lower=theta_l,  upper=theta_u, yvec=a$density)
```
